# Significance of the Neutrophil-to-Lymphocyte Ratio in p16-Negative Squamous Cell Carcinoma of Unknown Primary in Head and Neck

**DOI:** 10.3389/fonc.2020.00039

**Published:** 2020-01-29

**Authors:** Chunmiao Xu, Junhui Yuan, Wei Du, Junfu Wu, Qigen Fang, Xu Zhang, Hailiang Li

**Affiliations:** ^1^Department of Radiology, Affiliated Cancer Hospital of Zhengzhou University, Henan Cancer Hospital, Zhengzhou, China; ^2^Department of Head Neck and Thyroid, Affiliated Cancer Hospital of Zhengzhou University, Henan Cancer Hospital, Zhengzhou, China

**Keywords:** head neck squamous cell carcinoma, squamous cell carcinoma of unknown primary, neutrophil-to-lymphocyte ratio, prognosis, cancer cachexia

## Abstract

**Objective:** The neutrophil-to-lymphocyte ratio (NLR) has been reported to be associated with survival in solid malignancies. The main goal was to evaluate the prognostic significance of the NLR in patients with p16-negative squamous cell carcinoma of unknown primary (SCCUP) in head and neck.

**Methods:** The association between the NLR and clinical pathologic variables was evaluated by the chi-square test. The primary endpoint of interest was disease-specific survival (DSS). Univariate and Coxmodel analyses were used to evaluate prognostic factors.

**Results:** A total of 153 patients were included in the analysis. Cancer cachexia was noted in 10 patients. The mean NLR value was 3.9 (range: 1.4–8.3). A high NLR was significantly associated with cancer cachexia development. The 5-year DSS rate was 58%. In patients with NLRs varying from 1.4 to 3.7, the 5-year DSS rate was 71%; in patients with NLRs varying from 3.7 to 6.0, the 5-year DSS rate was 57%; in patients with NLRs varying from 6.0 to 8.3, the 5-year DSS rate was 39%, and the difference was significant (*p* = 0.001). Further Cox model analysis confirmed the independence of the NLR in predicting survival.

**Conclusions:** In patients with p16-negative SCCUP, an NLR ≥ 6.0 is significantly associated with worse prognosis.

## Introduction

Squamous cell carcinoma (SCC) of the head and neck (SCCHN) is one of the relatively uncommon malignant epithelial tumors in the body and usually has neck lymph node metastasis at initial diagnosis. However, in the clinic, ~1–4% of metastatic nodes can occur without any evidence of primary disease even after a clinical examination by a head and neck specialist, cytology and biopsy, endoscopy and radiological investigations including ultrasound, CT, MRI, and PET/CT (1). These lesions could be divided into two groups based on the result of immunohistochemical analysis of p16, p16-negative patients with squamous cell carcinoma of unknown primary (SCCUP) are more common and have significantly different survival compared with p16-positive patients (2, 3). There is great controversy regarding the optimal treatment of p16-negative patients (4–7), definite prognostic factors remain unclear (8–11).

Recent basic studies present the important role of the systemic inflammatory response in tumor cell invasion by promoting tumor cell proliferation, microvascular regeneration, and tumor metastasis (12, 13). The neutrophil-to-lymphocyte ratio (NLR) is a reliable and accurate marker of systemic inflammation and is closely related to the prognosis of SCC in the oral cavity, nasopharynx, oropharynx, larynx, and hypopharynx (14–17). Ulcer lesions are the most common manifestation of SCC in the head and neck. Systematic inflammatory responses might be more associated with primary disease than metastatic nodes (18), but whether there is also a significant relationship between prognosis and the NLR in p16-negative SCCUP remains unknown. Therefore, the main goal of the current study was to clarify these questions.

## Methods

### Ethical Considerations

The Zhengzhou University Institutional Review Board approved this retrospective study, and all patients had given written consent for medical research before initial treatment. All the procedures were consistent with the relevant requirements.

**Figure 1:**
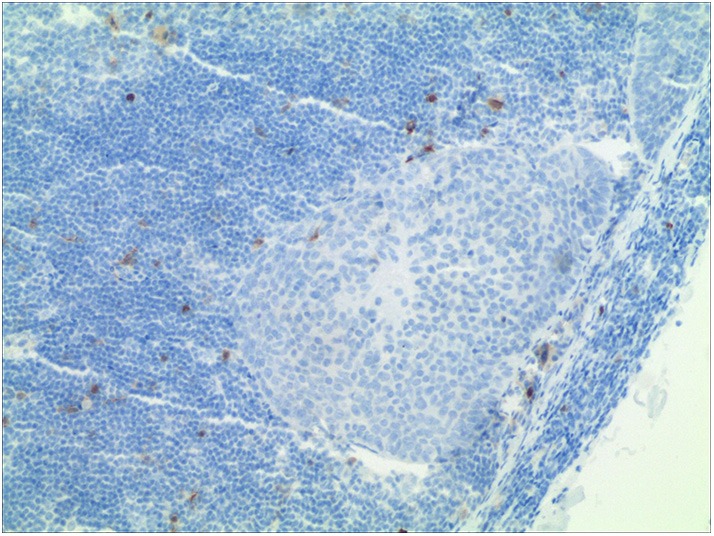
Negative result of p16 in immunohistochemical analysis (HE staining × 100).

From January 2000 to December 2016, the medical records of patients with surgically treated SCCUP were retrospectively reviewed. Enrolled patients had to meet the following criteria: first, no previous history of SCC of the head and neck; second, negative immunohistochemistry of p16 (Figure 1); and third, no autoimmune disease or regular use of glucocorticoids. Data regarding age, sex, node stage based on the AJCC 7th system, the NLR, operation record, pathology record, and follow-up were extracted and analyzed. All pathologic sections were re-reviewed by at least two pathologists, and if the status of p16 was unknown, an immunohistochemistry analysis was performed.

In our cancer center, investigations of clinical and endoscopic examination, ultrasound, CT or/and MRI, and PET-CT were always performed in patients with a neck mass after malignant disease was suggested by fine-needle aspiration biopsy. Neck dissection of level I-V was suggested for all patients, bilateral operation was performed if there was doubt of bilateral lesion. Functional neck dissection was usually the first choice unless there was N3 disease or apparent invasion of sternocleidomastoid muscle or internal jugular vein or accessory nerve. After therapy, the patients were examined every 3 months during the first year, every 6 months during the second year, and once per year after the second year.

The NLR was calculated by dividing the absolute neutrophil count by the absolute lymphocyte count within 2 weeks before operation (14–17, 19, 20). The optimal cut-off for the NLR remains unclear, and previous authors have reported that the cut-off varies from 1.58 to 5.0 according to the mean, median, tertile value, and ROC curve. In the current study, the cut-off was formulated based on the tertile value. Cancer cachexia was defined as an unintentional weight loss of at least 5% premorbid weight occurring over the course of 3–6 months (21).

Patient demographic variables were compared across NLR values using the chi-square test. The primary endpoint of interest was disease-specific survival (DSS), and the survival time was calculated from the date of surgery to the event or the last follow-up. The Kaplan-Meier method (log-rank test) was used to plot the DSS curve. The factors of age, sex, smoking, drinking, neck lymph node stage, extracapsular spread, cancer cachexia, adjuvant treatment, and NLR were included for analyzing their association with DSS, and the variables that were significant in the univariate analysis were then analyzed in the Cox model to find the independent prognostic factors with the use of a forward stepwise (likelihood ratio) procedure. All statistical analyses were performed using SPSS 20.0, and *p* < 0.05 was considered significant.

## Results

A total of 153 patients (121 male and 32 female) were enrolled for analysis, and the mean age was 62.7 (range: 28–73) years. Smoking and drinking status were reported in 57 and 43 patients, respectively. Cancer cachexia was noted in 10 patients. Thirty-one patients presented with pN1 disease, 53 patients with pN2a disease, 61 patients with pN2b disease, 6 patients with pN2c disease, and 2 patients with pN3 disease. Extracapsular spread (ECS) was reported in 37 patients. Disease grade was distributed as follows: well differentiation in 20 patients, moderate differentiation in 73 patients, and poor differentiation in 60 patients. The mean NLR value was 3.9 (range: 1.4–8.3).

Unilateral neck dissection was performed in 147 patients, and bilateral neck dissection was performed in 6 patients. Radical neck dissection was performed in 15 patients, modified radical neck dissection was performed in 84 patients, and functional neck dissection was performed in 60 patients.

The associations between the NLR and clinical pathologic variables are presented in Table 1. A high NLR value was significantly related to the presence of cachexia (*p* = 0.008). No significant relationship between the NLR and other variables was noted (all *p* > 0.05).

**Table 1:** Association between neutrophil-to-lymphocyte ratio (NLR) and clinical pathologic variables in patients with squamous cell carcinoma of unknown primary.

	**NLR**			***p***
**Variables**	**1.4 ≤ NLR < 3.7**	**3.7 ≤ NLR < 6.0**	**6.0 ≤ NLR ≤ 8.3**	
	**(*n* = 53)**	**(*n* = 67)**	**(*n* = 33)**	
Age				
≤62	24	30	15	
>62	29	37	18	0.997
Sex				
Male	40	54	27	
Female	13	13	6	0.719
Smoker				
Yes	18	24	15	
No	35	43	18	0.534
Drinker				
Yes	15	20	8	
No	38	47	25	0.841
Cancer cachexia				
Yes	1	3	6	
No	52	64	27	0.008
Neck node stage				
N1 + 2a	31	39	14	
N2b + 2c + 3	22	28	19	0.266
Disease grade				
Well	7	10	3	
Moderate	25	33	15	
Poor	21	24	15	0.882
ECS^*^				
Yes	11	15	11	
No	42	52	22	0.374

* *ECS, Extracapsular spread. Significant variables are in bold*.

The mean follow-up time was 79.5 (range: 15–221) months, 138 patients underwent adjuvant radiotherapy, and 64 patients underwent adjuvant chemotherapy. Recurrence occurred in 85 patients: 69 patients had loco-regional recurrence, and 16 patients had simultaneous loco-regional and distant recurrence. In these 85 patients, 14 cases received a second operation, 43 cases were treated by palliative chemotherapy combined with targeted drug, 28 cases refused any treatment.

**Figure 2:**
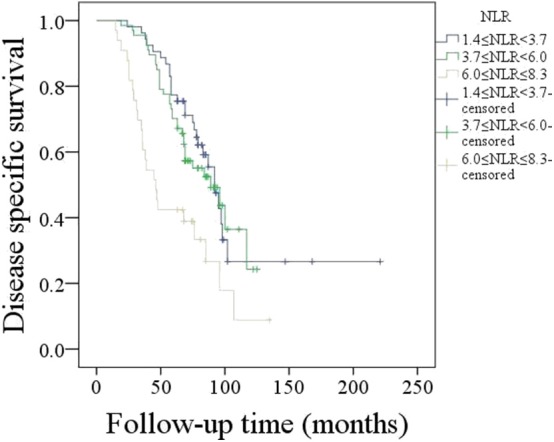
Disease-specific survival in patients with different neutrophil-to-lymphocyte ratios (NLRs) (*p* = 0.001).

A total of 73 patients died of the disease, and the 5-year DSS rate was 58%. In patients with an NLR varying from 1.4 to 3.7, the 5-year DSS rate was 71%; in patients with an NLR varying from 3.7 to 6.0, the 5-year DSS rate was 57%; in patients with an NLR varying from 6.0 to 8.3, the 5-year DSS rate was 39%, and the difference was significant (Figure 2, *p* = 0.001). Further Cox model analysis confirmed the independence of the NLR in predicting survival (Table 2).

**Table 2:** Prognostic factors for disease specific survival in patients with squamous cell carcinoma of unknown primary.

	**Univariate**	**Cox model**	
**Variables**	**Log-rank test**	**HR (95% CI)**	***p***
Age	0.321		
Sex	0.143		
Smoker	0.032		
Drinker	0.532		
Lymph node stage (N1 +	0.004	5.83 (2.91–16.31)	<0.001
2a vs. N2b + 2c + 3)			
ECS^*^	0.006	3.65 (1.47–8.44)	0.003
Cancer cachexia	0.021	2.12 (1.75–7.16)	0.001
Radiotherapy	0.423		
Chemotherapy	0.218		
NLR^%^ 1.4 ≤ NLR < 3.7	0.001		
3.7 ≤ NLR < 6.0		1.67 (0.45–2.68)	0.416
6.0 ≤ NLR ≤ 8.3		2.54 (1.62–4.05)	0.011

* *ECS, Extracapsular spread; ^%^NLR, neutrophil-to-lymphocyte ratio. Significant variables are in bold*.

## Discussion

The role of the NLR in predicting prognosis in SCCHN has been widely discussed (15–17, 22). Rachidi et al. (16) reported that patients with oral, pharyngeal, and laryngeal cancers in the lowest tertile of the NLR were at a lower risk for worse prognosis compared with those in the highest tertile after multivariate analysis. Moreover, the NLR was lower in patients with HPV-positive tumors compared to the NLR in patients with HPV-negative tumors, and the NLR predicted survival in both tumor types. Rassouli et al. (17) described that the subgroup with an NLR ≤ 3.0 was associated with higher T classification and had the highest mortality, whereas an NLR > 4.2 predicted higher rates of recurrence. A recent meta-analysis concluded that an elevated NLR was predictive of poorer overall survival in patients with HNSCC (15). Our previous research also showed that a high NLR was associated with worse prognosis in locally advanced tongue SCC (20). However, none of the abovementioned studies evaluated the p16-negative SCC of unknown primary; thus, this study was the first to note that compared with patients in the other two subgroups, patients with an NLR > 6.0 had the worst prognosis. It would be of great benefit to identify HNSCC patients who display aggressive biological behaviors at the time of diagnosis and then to provide improved communication with the patient preoperatively regarding their future possible prognosis.

The association between the NLR and clinical pathologic variables has been described by previous studies in head and neck cancer (19, 20, 23). Our previous studies reported that a high NLR was related to advanced disease stage, poor differentiation, and advanced lymph node stage in parotid cancer (19, 20). Kano et al. (23) demonstrated significant relationships between a high NLR and oropharyngeal or hypopharyngeal cancer, T3 to T4, N2b to N3, and clinical stage III to IV. However, in the current study, we only found that a high NLR was associated with cancer cachexia and not the abovementioned factors. This finding was interesting. The underlying mechanism might be associated with some signaling pathways. It has been well-established that adipocytes play a relevant role in the establishment of white adipose tissue inflammation through the activation of the NF-κB and inflammasome pathways (24). Studies of obese patients showed increased activity of the NF-κB pathway in adipose tissue and, consequently, local inflammation (25). The proinflammatory cytokines IL-1β and IL-18 are synthesized as immature proteins and require the inflammasome pathway for cleavage and release, yielding their mature, biologically active cytokine forms (26). Our future work will focus on the relationship between the NLR and these established inflammasome pathways for valuable clarification of cancer cachexia.

It is still unclear whether the NLR plays the role of a non-specific marker of systemic stress that is associated with poor outcomes and whether the systemic inflammatory response is a reflection of a tumor that is unresponsive to treatment, similar to performance status. The exact association between a high NLR and poor outcome in cancer patients has not been fully elucidated. Some possible explanations can be mentioned based on previous evidence. The inflammatory microenvironment induces genotoxic stress via multiple mechanisms, including reactive oxygen species and the induction of activation-induced cytidine deaminase. The pretreatment NLR value reflects the status of the immune system and systemic inflammation. Elevation of neutrophils is a sign of local as well as systemic inflammatory responses. Several cytokines and angiogenic factors are produced by neutrophils, and these agents play an important role in promoting tumor development (27). Additionally, hematological markers might be surrogate markers of cancer cachexia, which is related to poor survival (28, 29). On the other hand, lymphocytes are associated with immune surveillance and act by eliminating cancer cells (30). Therefore, a high NLR is considered to predict a worse prognosis.

Cancer cachexia is mainly caused by diminished oral intake and catabolic factors secreted by tumors, including interferon-gamma, interleukins, and tumor necrosis factor (31). A recent review demonstrated that cancer cachexia had a prevalence ranging from 20.2 to 32.2% in SCCHN (32). Orell-Kotikangas et al. (33) reported the presence of cachexia in 31% of 65 patients with SCCHN. A higher risk for cancer cachexia was expected in SCCHN patients, owing to the reduced food intake in these patients. However, the incidence of SCCUP remains unknown. As mentioned above, food intake is significantly adversely affected by swallowing-related symptoms, such as pain and dysphagia, caused by primary tumors. Therefore, SCCUP patients might have unique characteristics regarding cancer cachexia. The incidence of cachexia was noted to be just 6.5% in our study, which is considerably lower than the results described above. The main reason for this finding was the lack of a primary tumor responsible for cachexia development.

Prognosis in SCCUP has been frequently analyzed, and widely accepted risk factors include advanced node stage, extracapsular spread, and adjuvant therapy (31–34). Similar findings were also noted in the current study. Moreover, we were the first to find that cancer cachexia significantly decreased prognosis. Both Orell-Kotikangas et al. (33) and Kwon et al. (34) reported that cachexia was related to a higher probability of non-cancerous death, cancer-specific death, and overall death in patients with SCCHN. Our 5-year DSS rate was slightly lower than previous data (35), possibly because only p16-negative patients were enrolled, and a p16-negative status usually indicates poor survival (36).

The limitations of this study must be acknowledged. First, it was a retrospective study, and the sample size was relatively small. A larger sample size in a prospective study is thus needed to clarify these questions. Second, although the NLR has been well-established as an indicator of prognosis in a variety of cancers, including breast, esophageal, renal, and liver cancers, the complexity of carcinogenesis is so high in malignancies that a single indicator may not be a reliable predictor to determine outcome; additionally, neutrophil and lymphocyte counts are non-specific parameters because they can be influenced by concomitant conditions, such as infections or inflammation.

In summary, the NLR is significantly associated with prognosis in patients with p16-negative SCCUP.

## Data Availability Statement

All data generated or analyzed during this study are included in this published article. And the primary data could be obtained from the corresponding author.

## Ethics Statement

The Zhengzhou University institutional research committee approved our study and all participants signed an informed consent agreement. All the related procedures were consistent with Ethics Committee regulations.

## Consent to Publish

All the material came from our cancer center, and the publish consent have been obtained from all the patients.

## Author Contributions

CX, HL, WD, and QF: study design, manuscript writing, and manuscript revising: JY, WD, and QF: studies selecting and data analysis. JY, JW, XZ, and QF: study quality evaluating. All authors have read and approved the final manuscript.

### Conflict of Interest

The authors declare that the research was conducted in the absence of any commercial or financial relationships that could be construed as a potential conflict of interest.
